# Supplementary material for: Interdependence of Pasha and Drosha for localization and function of the Microprocessor in C. elegans
Source: Nat Commun. 2025 Jul 1;16:5595. doi: 10.1038/s41467-025-60721-5 (PMC12219410; doi:10.1038/s41467-025-60721-5)
Supplement: Supplementary file 1 — Supplementary Information [file 41467_2025_60721_MOESM1_ESM.pdf]

Supplementary Information

Interdependence of Pasha and Drosha for localization and function of the Microprocessor in *C. elegans*

Thiago L. Knittel[1,#], Brooke E. Montgomery[1,#], Kailee J. Reed[1,2], Madeleine C. Chong[1], Ida J. Isolehto[3,4], Erin R. Cafferty[1], Margaret J. Smith[1], Reese A. Sprister[1], Colin N. Magelky[1], Hataichanok Scherman[5], Rene F. Ketting[3,6], and Taiowa A. Montgomery[1,2*]

[1]Department of Biology, Colorado State University, Fort Collins, CO 80523, USA

[2]Cell and Molecular Biology Program, Colorado State University, Fort Collins, CO 80523, USA

[3]Biology of Non-coding RNA group, Institute of Molecular Biology, Mainz, Germany

[4]International PhD Program on Gene Regulation, Epigenetics and Genome Stability, Mainz, Germany

[5]Department of Biochemistry and Molecular Biology, Colorado State University, Fort Collins, CO 80523, USA

[6]Institute of Developmental Biology and Neurobiology, Johannes Gutenberg University, Mainz, Germany

[#]Equal contribution

*Correspondence: tai.montgomery@colostate.edu

SUPPLEMENTARY METHODS

Strains

The *ubl-1::mCherry::pri-mir-58-sensor-mut* mutation was introduced into the CMP1 *ubl-1::mCherry::pri-mir-58-sensor* plasmid using NEB Q5 site directed mutagenesis (New England Biolabs, E0554S) with the primers GGGATGAGATTGTTCAGTACG and TATGGTATTGGACGAAGTG. To generate *FLAG::pash-1(ram39*[149-266]*)*, the corresponding *pash-1* gene fragment was PCR amplified from cDNA with primers extending the 5' end with a 3xFLAG peptide sequence and the 5' and 3' ends with pDONR entry vector compatible sequences (GGGGACAACTTTTCTATACAAAGTTGACATGGATTATAAAGACGATGACGATAAGCGTGACTACAAGGACGACGACGACAAGCGTGATTACAAGGATGACGATGACAAGAGTGTCGGTGAACAAATTCG and GGGGACAACTTTATTATACAAAGTTGTCGGTGACATTCGAAGCTCCTTGAC). The resulting fragment was cloned into pDONR 221 P4r-P3r and then recombined into CMP1 with the *ubl-1* regulatory sequences described in main text. A stop codon within the *ubl-1* 3'UTR was utilized to terminate translation, extending the C terminus of 3xFLAG::PASH-1[149-266] protein by 22 amino acids. The resulting plasmid was transformed into wild-type (N2) animals using Mos1-mediated single copy insertion. The SV40-NLS and EGL-13-NLS encoding sequences were inserted into *pash-1::GFP* and *mCherry::drsh-1* in the various strains described in separate injection steps using CRISRP-Cas9-mediated genome editing with purified Cas9 protein, synthesized guide RNAs (*pash-1 SV40-NLS:* UCAUAUGUUUGUUGUUUUGU, *pash-1 egl-13-NLS:* CAGAAACCACACAAAAGUAA, *drsh-1 SV40-NLS*: UUCAUUUAGAUGGUCUCAAA, and *drsh-1 egl-13-NLS*: CAUGGAUGAAUUGUAUAAGU), and repair templates (*pash-1 SV40-NLS*: CTGGGATTACACATGGCATGGATGAACTATACAAACCAAAGAAGAAGCGTAAGGTCTAGTATATTCACCTCATATGTTTGTTGTTTTGTTGCTAGTTTTAATTTTTAAATATGTATTGTTTAGAAAT, *pash-1 egl-13-NLS:* ATTCATCATCCCCAAGTCATCAGAAACCACACAAATCCCGTCGTCGTAAGGCCAACCCAACCAAGCTCTCCGAGAACGCCAAGAAGCTCGCCAAGGAGGTCGAGAACAGTAAAGGAGAAGAACTTTTCACTGGAGTTGTCCC, *drsh-1 SV40-NLS*: GAAATTGTAGACAGATTTAGATTTTCATTTAGATGCCAAAGAAGAAGCGTAAGGTCGTCTCAAAGGGTGAAGAAGATAACATGGCAATTAT, and *drsh-1 egl-13-NLS:*

GGCACTCGACAGGTGGCATGGATGAATTGTATAAGTCCCGTCGTCGTAAGGCCAACCCAACCAAGCTCTCCGAGAACGCCAAGAAGCTCGCCAAGGAGGTCGAGAACTCGGACGAAAAGATTTCAATGACGCTTAACTTCCC) (Integrated DNA Technologies). SV40-NLS encoding sequence (CCAAAGAAGAAGCGTAAGGTC) was inserted after the last codon of GFP in *pash-1::GFP* strains and directly after the start codon in *drsh-1::mCherry* strains. EGL-13-NLS encoding sequence (TCCCGTCGTCGTAAGGCCAACCCAACCAAGCTCTCCGAGAACGCCAAGAAGCTCGCCAAGGAGGTCGAGAAC) was inserted before the first GFP codon in *pash-1::GFP* strains and after the last mCherry codon in *drsh-1::mCherry* strains. See Supplemental Data 3 for strain names and genotypes.

SUPPLEMENTARY FIGURES

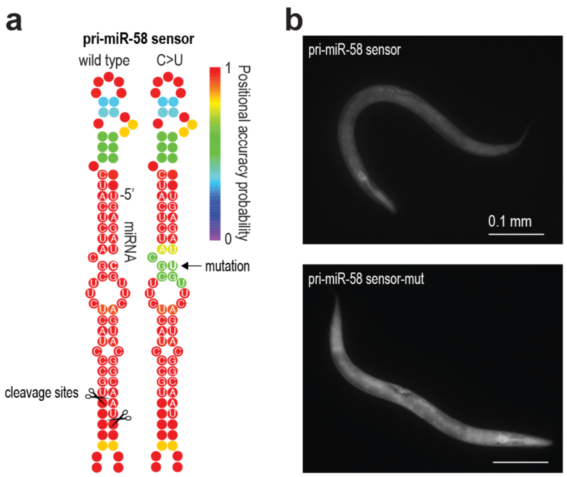

Supplementary Fig. S1. A mutation in the hairpin partially desilences the pri-miR-58 sensor. **a** Secondary structure predictions of wild type and C-U mutant miR-58 hairpins. Nucleotide sequence is shown for the miRNA duplex region. The 5' end of the miRNA is indicated. The cleavage sites are indicated with scissors. **b** mCherry fluorescence in animals containing the wild-type (pri-miR-58 sensor) or C-U mutant (pri-miR-58 sensor-mut) construct. Two representative L4 stage animals were imaged for each strain.

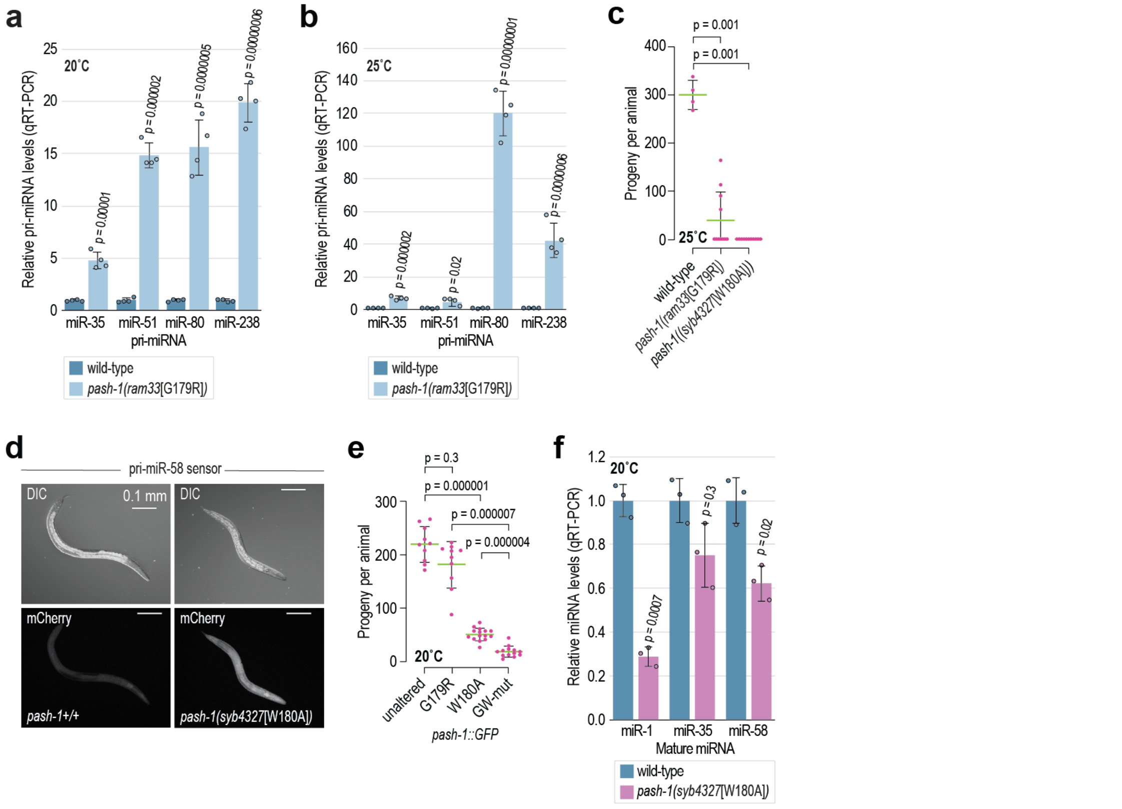

Supplementary Fig. S2. Impact of PASH-1 G179R and W180A mutations on miRNA processing.
a, b Relative levels of several pri-miRNAs in wild-type and *pash-1(ram33*[G179R]*)* mutants grown at 20˚C (**a**) or 25˚C (**b**) as determined by SYBR Green qRT-PCR. Data is normalized to *rpl-32* mRNA levels. Error bars are SD. n = 4 biological replicates. Two-tailed, two-sample Student's t-tests were used to calculate *p*-values for comparisons to wild-type. A Bonferroni correction for four comparisons was applied to each. **c** Numbers of progeny produced by wild-type, *pash-1(ram33*[G179R]*),* and *pash-1(syb4327*[W180A]*)* animals grown at 25˚C. Error bars are SD. n = 4 (wild-type) or 11 (p*ash-1*(*ram33*[G179R]*)* and *pash-1(syb4327*[W180A])) animals. *p*-values were calculated using two-tailed Mann-Whitney U tests. **d** Images showing mCherry fluorescence in control (*pash-1+/+*) and *pash-1(syb4327*[W180A]*)* animals containing the pri-miR-58 sensor construct. Three representative animals were imaged for each genotype. **e** Numbers of progeny produced by *pash-1::GFP, pash-1*[G179R]*::GFP, pash-1*[W180A]*::GFP,* and *pash-1*[GW-mut]*::GFP* animals grown at 20˚C. Error bars are SD. n = 10 (unaltered and G179R), 13 (GW-mut), or 15 (W180A) animals. *p*-values were calculated using two-tailed Mann-Whitney U tests. **f** Relative levels of miR-1, miR-35, and miR-58 in wild-type and *pash-1(syb4327*[W180A]*)* grown at 20˚C as determined by TaqMan qRT-PCR. Data is normalized to 22G-rRNA levels. Error bars are SD. n = 3 biological replicates. Two-tailed, two-sample Student's t-tests were used to calculate *p*-values for comparisons to wild-type. A Bonferroni correction for three comparisons was applied. Source data are provided as a Source Data file.

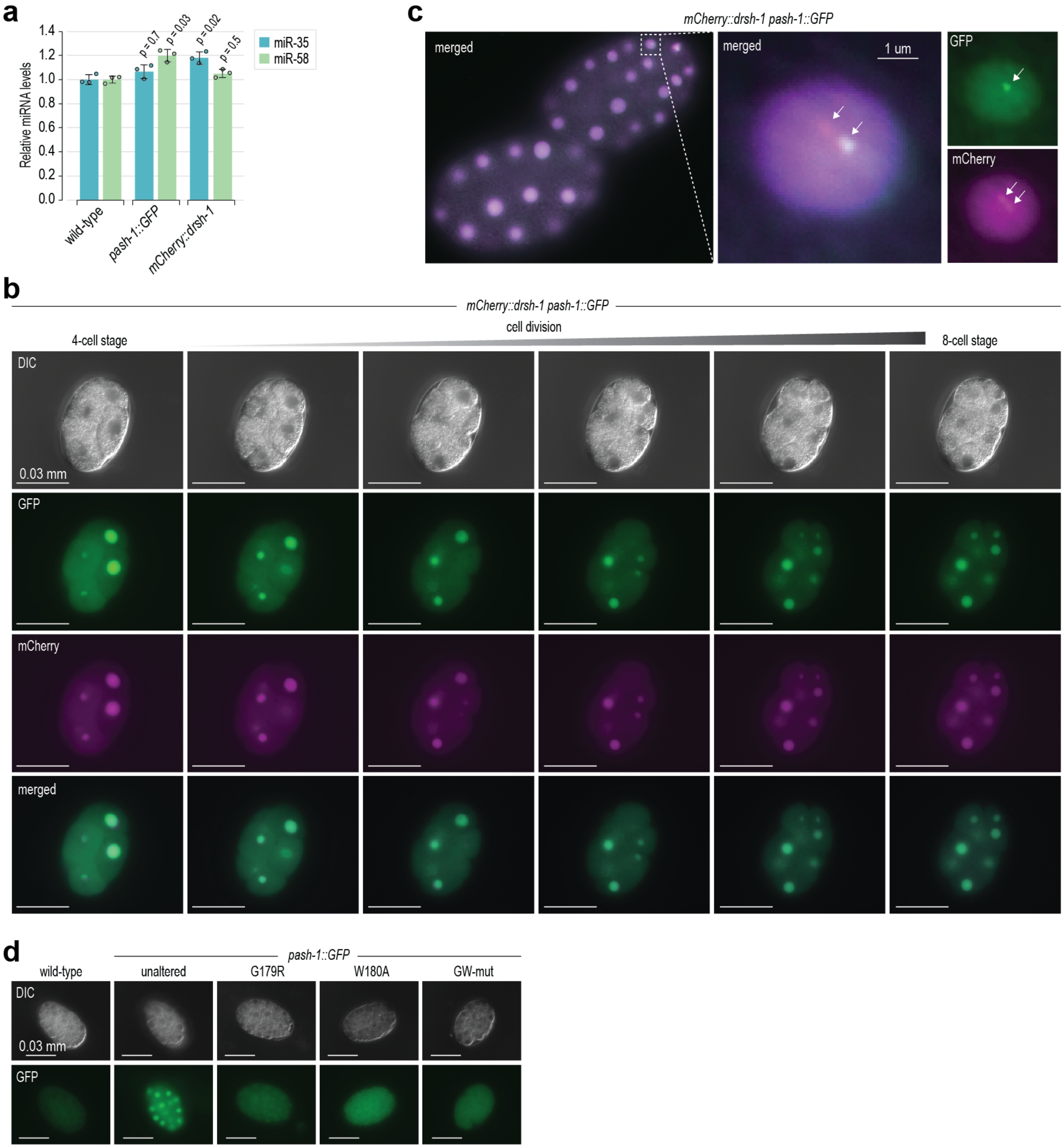

Supplementary Fig. S3. Subcellular localization of PASH-1 and DRSH-1. **a** Relative levels of miR-35 and miR-58 in wild-type, *pash-1::GFP*, and *mCherry::drsh-1* animals grown at 20˚C as determined TaqMan qRT-PCR. Data is normalized to 21UR-1 piRNA levels. Error bars are SD. n = 3 biological replicates. Two-tailed, two-sample Student's t-tests were used to calculate *p*-values for comparisons to wild-type. A Bonferroni correction for two comparisons was applied to each. **b** PASH-1::GFP and mCherry::DRSH-1 localization during cell divisions taking place between the 4-cell and 8-cell stage. A single embryo was imaged across the entire stage series. **c** PASH-1::GFP and mCherry::DRSH-1 nuclear localization in a single nucleus from an embryo. Arrows point to nuclear foci. Sixteen embryos were imaged for each strain. **d** GFP fluorescence in wild-type, *pash-1::GFP* (unaltered), *pash-1*[G179R]*::GFP*, *pash-1*[W180A]*::GFP*, and *pash-1*[GW-mut]*::GFP* embryos. Three representative embryos were imaged for each strain. Source data are provided as a Source Data file.

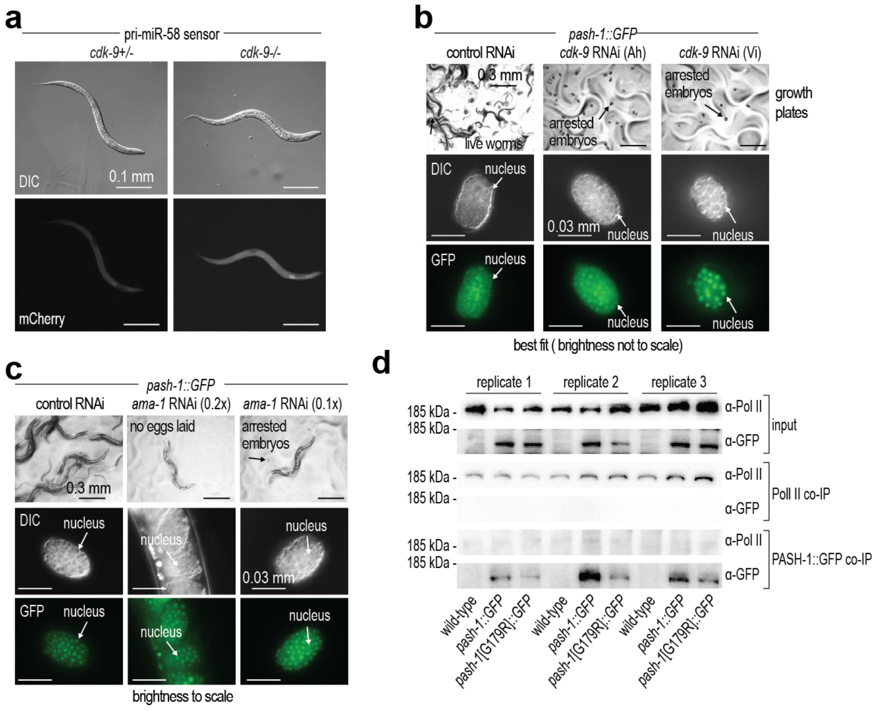

Supplementary Fig. S4. Impact of reduced RNA Polymerase II activity on PASH-1::GFP nuclear localization. **a** mCherry fluorescence in *cdk-9(tm2884)+/-* and *cdk-9(tm2884)-/-* animals containing the pri-miR-58 sensor. At least eleven animals were imaged for each genotype. **b** PASH-1::GFP localization in control (empty L4440 vector) or *cdk-9* RNAi-treated animals. Two different *cdk-9* RNAi clones were used (Ah, Ahringer; Vi, Vidal). At least 4 embryos were imaged for each condition. Images of growth plates showing embryonic arrest caused by *cdk-9* knockdown are also shown. **c** PASH-1::GFP localization in control (L4440) or *ama-1* RNAi-treated animals. Two different concentrations of the *ama-1* RNAi clone were used (0.1x or 0.2x, diluted with L4440 vector control bacteria). Images of growth plates showing the lack of deposited embryos on the plate (0.2x) or arrested embryos (0.1x) caused by *ama-1* knockdown are also shown. At least three embryos were imaged for each condition. **d** Western blot analysis of PASH-1::GFP or PASH-1[G179R]::GFP and Pol II co-IPs. n = 3 biological replicates. Source data are provided as a Source Data file.

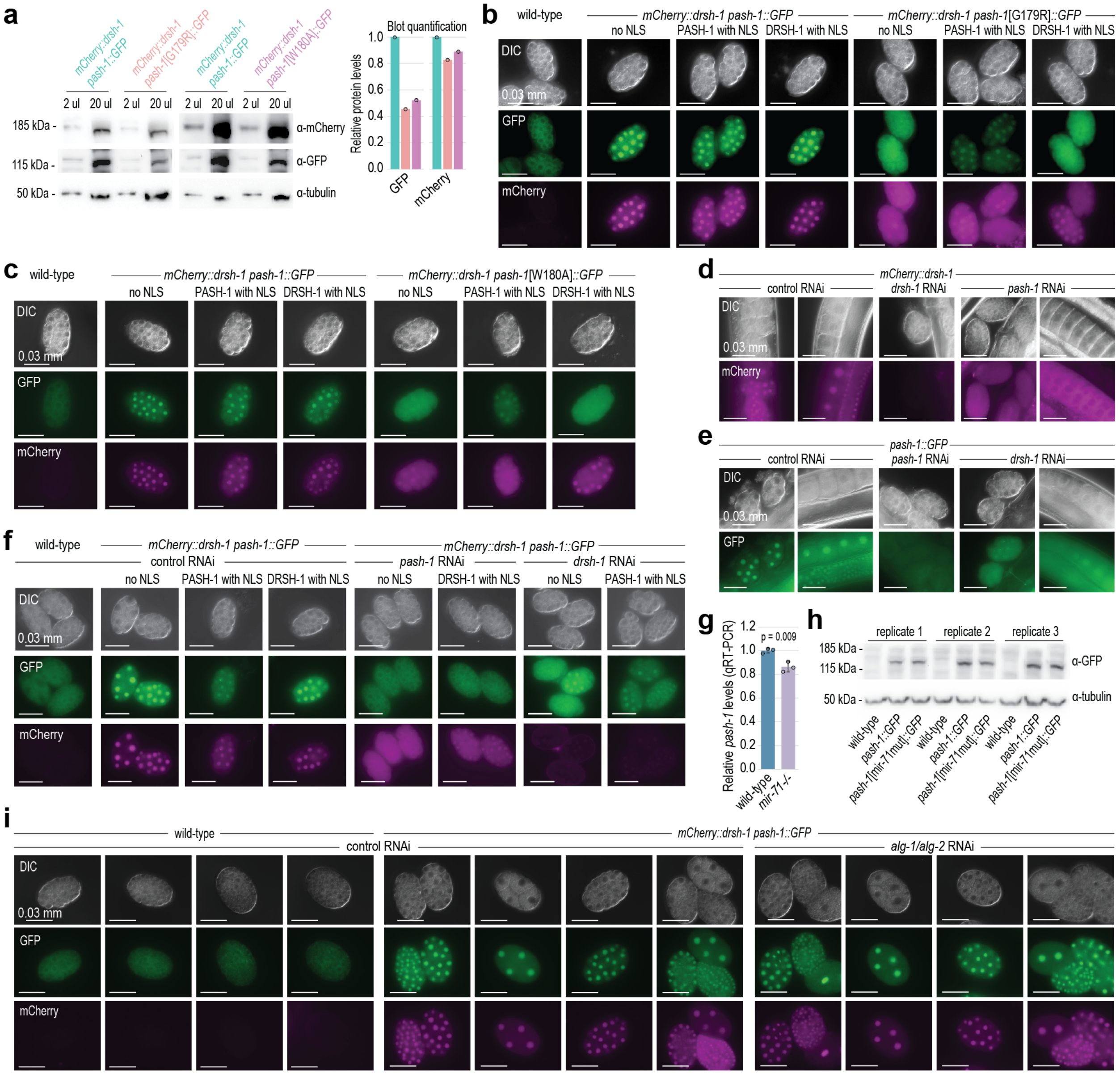

Supplementary Fig. S5. Interdependency of PASH-1 and DRSH-1 for proper nuclear localization. **a** mCherry::DRSH-1 and wild-type and mutant PASH-1::GFP protein levels as determined by Western blot. Tubulin is shown as a loading control. Quantification of GFP and mCherry levels are shown to the right with normalization to tubulin and relative to *mCherry::drsh-1 pash-1::GFP* controls. n = 1 biological replicate at 2 volumes. Green bar: *mCherry::drsh-1 pash-1::GFP*. Salmon bar: *mCherry::drsh-1 pash-1*[G179R]*::GFP*. Purple bar: *mCherry::drsh-1 pash-1*[W180A]*::GFP*. **b, c** GFP and mCherry fluorescense in wild-type, *mCherry::drsh-1 pash-1::GFP* and *mCherry::drsh-1 pash-1*[G179R]*::GFP* (**b**) or *mCherry::drsh-1 pash-1*[W180A]*::GFP* (**c**) embryos with 0 or 2 ectopic NLSs on PASH-1 or DRSH-1. At least twelve embryos were imaged for each strain. **d, e** mCherry::DRSH-1 (**e**) or PASH-1::GFP (**f**) localization in germlines and embryos of control (L4440), *drsh-1*, and *pash-1* RNAi-treated animals. Animals were grown for 72 hours at 20˚C on the indicated RNAi treatments. Embryos or germlines from at least two individuals were imaged for each condition. **f** GFP and mCherry fluorescense in wild-type and *mCherry::drsh-1 pash-1::GFP* embryos with DRSH-1 or PASH-1 containing two ectopic NLSs following control (L4440), *pash-1*, or *drsh-1* RNAi. At least ten embryos were imaged for each condition. **g** Relative *pash-1* mRNA levels in wild-type and *mir-71-/-* mutant adult animals as determined by qRT-PCR. *rpl-32* mRNA levels were used for normalization. Error bars are SD. n = 3 biological replicates. A two-tailed, two-sample Student's t-test was used to calculate the *p*-value. **h** Levels of PASH-1 protein produced from *pash-1::GFP* and *pash-1*[mir-71mut]*::GFP* as determined by Western blot. n = 3 biological replicates. Tubulin is shown as a loading control. **i** PASH-1::GFP and mCherry::DRSH-1 expression in embryos following control (L4440) or *alg-1/alg-2* RNAi. At least 2 embryos were imaged for each condition. Source data are provided as a Source Data file.

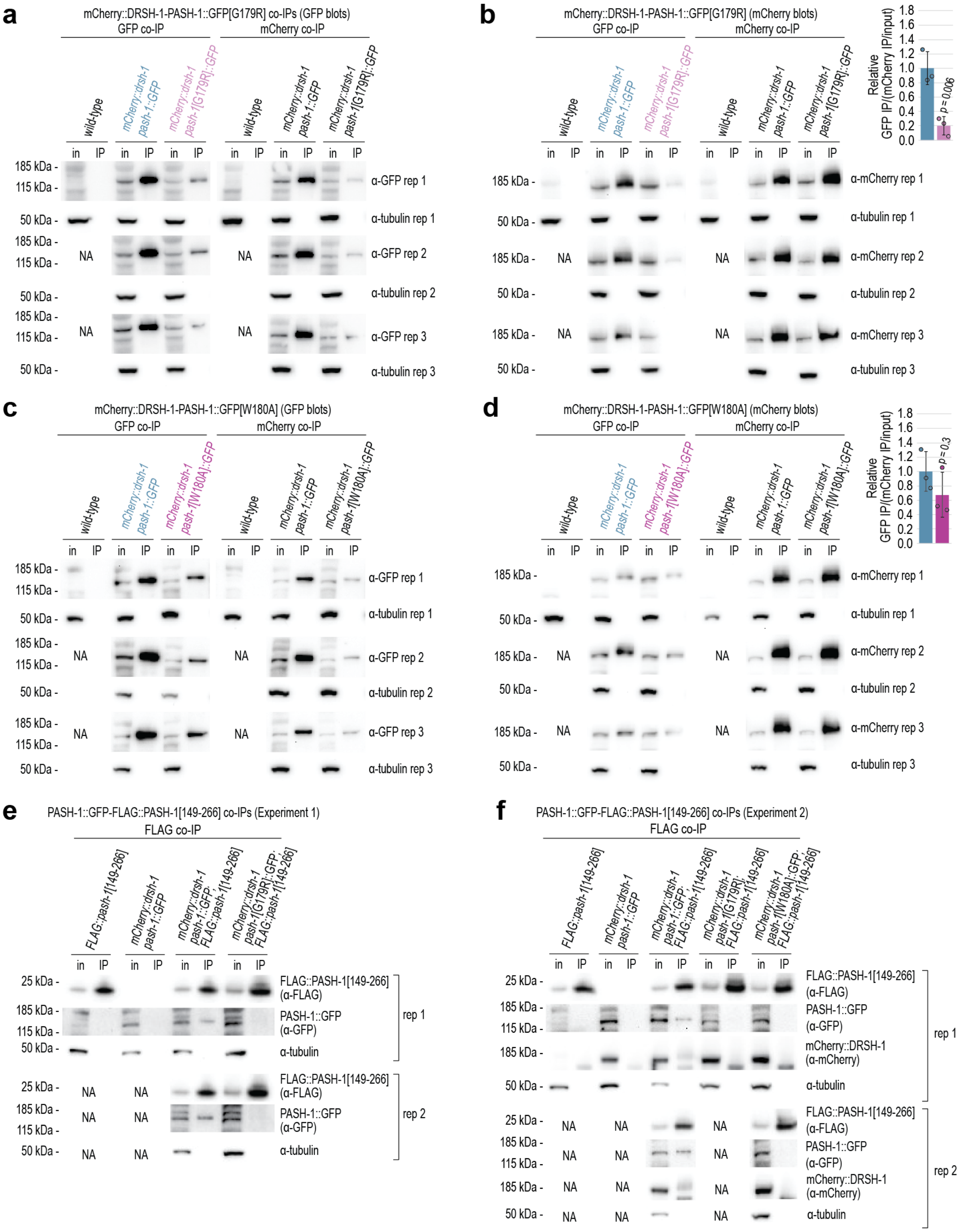

Supplementary Fig. S6. Microprocessor assembly in PASH-1 GW motif mutants. **a-f** Western blot analysis of DRSH-1-PASH-1 interactions. Tubulin is shown as a loading control. n = 1-3 biological replicates, as indicated. in, cell lysate input fraction; IP, co-IP fraction. **a, b** Western blot analysis of PASH-1::GFP and either PASH-1[G179R]::GFP (**a**) or mCherry::DRSH-1(**b**) co-IP'd with GFP or mCherry antibodies. Ratio of IP to input sample loaded = 9:1. The plot in (**b**) shows the mean ratio of GFP levels in IP fractions relative to mCherry levels in IP fractions normalized to mCherry levels in input fractions. Values are relative to the *mCherry::drsh-1 pash-1::GFP* control. A two-sample Student's t-test was used to calculate the *p*-value. **c, d** As in (**a, b**) but with PASH-1[W180A]::GFP instead of PASH-1[G179R]::GFP. **e, f** Western blot analysis of PASH-1::GFP, PASH-1[G179R]::GFP, PASH-1[W180A]::GFP, and mCherry::DRSH-1 co-IP'd with FLAG::PASH-1[149-266] in two independent experiments. n = 1-2 biological replicates, as indicated. Ratio of IP to input sample loaded was 20:1 but in these blots, 2.5x protein equivalents were loaded for the G179R and W180A co-IPs to make them directly comparable to unaltered PASH-1::GFP. Proteins were co-IP'd with FLAG antibody. Source data are provided as a Source Data file.

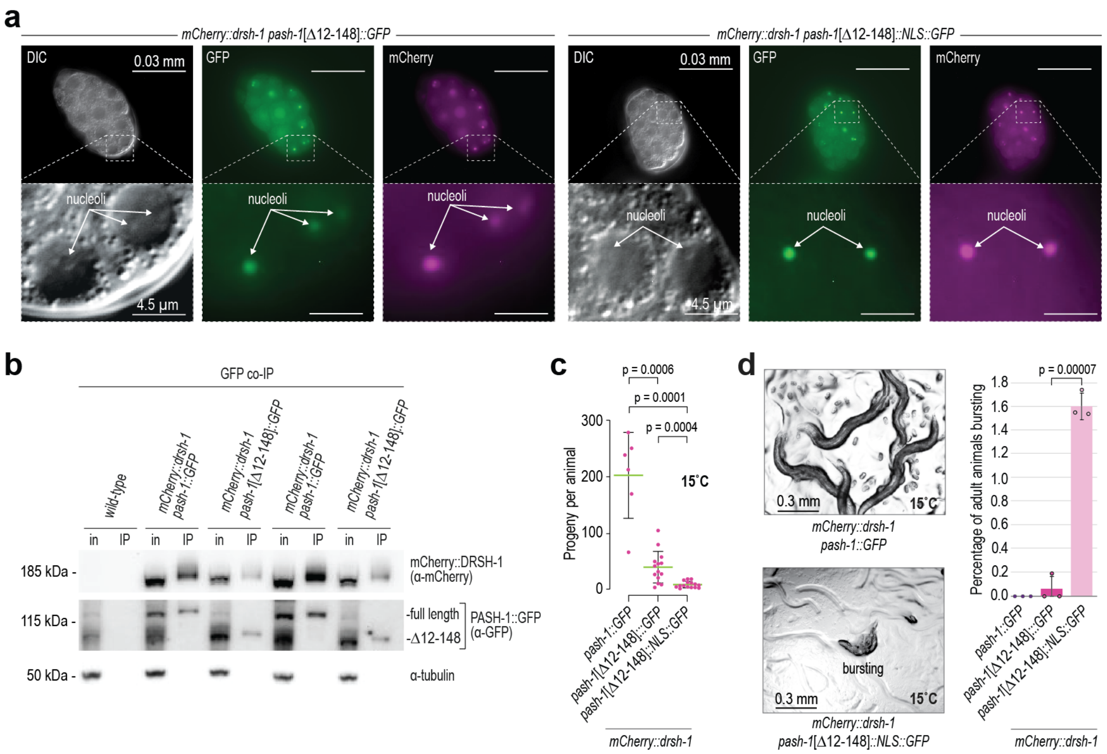

Supplementary Fig. S7. Impact of deleting PASH-1's N terminus. **a** GFP and mCherry fluorescense in *mCherry::drsh-1 pash-1*[Δ12-148]*::GFP* (12 embryos imaged) and *mCherry::drsh-1 pash-1*[Δ12-148]*::NLS::GFP* (9 embryos imaged) embryos from animals grown at 15˚C. **b** Western blot analysis of PASH-1::GFP, PASH-1[Δ12-148]::GFP, and mCherry::DRSH-1 co-IP'd using GFP antibody. The ratio of IP to input sample loaded was 20:1. in, input fraction from cell lysates; IP, co-IP fraction. Tubulin is shown as a loading control. Data from two biological replicates are shown, except for wild-type, with only 1 replicate. **c** Numbers of progeny produced by *mCherry::drsh-1 pash-1::GFP* (control), *mCherry::drsh-1 pash-1*[Δ12-148]*::GFP*, and *mCherry::drsh-1 pash-1*[Δ12-148]*::NLS::GFP* animals grown at 15˚C. Error bars are SD. n = 6 (control), 14 (*mCherry::drsh-1 pash-1*[Δ12-148]*::GFP*), or 15 (*mCherry::drsh-1 pash-1*[Δ12-148]*::NLS::GFP*) individuals. *p*-values were calculated using two-tailed Mann-Whitney U tests. **d** Representative images of *mCherry::drsh-1 pash-1::GFP* (grown for 6 days) and *mCherry::drsh-1 pash-1*[Δ12-148]*::NLS::GFP* animals (grown for 8 days). The bar plot shows the percentage of burst animals (n = 146-628 per replicate). Bursting was assessed in gravid adults after 6 days (control, 8 individuals imaged), 7 days (*pash-1*[Δ12-148]*::GFP*), or 8 days (*pash-1*[Δ12-148]*::NLS::GFP*, 22 individuals imaged) of growth at 15˚C. The *p*-value was calculated using a two-tailed, two-sample Student's t-test. Error bars are SD. n = 3 biological replicates, each with ≥146 animals assessed. Source data are provided as a Source Data file.
